# The Effect of Microstructural Changes Produced by Heat Treatment on the Electromagnetic Interference Shielding Properties of Ti-Based MXenes

**DOI:** 10.3390/nano15090676

**Published:** 2025-04-29

**Authors:** Xue Han, Jae Jeong Lee, Ji Soo Kyoung, Yun Sung Woo

**Affiliations:** 1 Department of Materials Science and Engineering, Dankook University, Dandae-ro 119, Dongnam-gu, Cheonan-si 31116, Chungnam-do, Republic of Korea; axh1999a@dankook.ac.kr (X.H.); jaejeong.lee@dankook.ac.kr (J.J.L.); 2 Department of Physics, Dankook University, Dandae-ro 119, Dongnam-gu, Cheonan-si 31116, Chungnam-do, Republic of Korea; kyoungjs@dankook.ac.kr

**Keywords:** Ti-based MXene, heat treatment, porous structure, EM absorption, EMI shielding effectiveness

## Abstract

Ti-based MXenes such as Ti_3_C_2_T_X_ and Ti_2_CT_X_ have attracted considerable attention because of their superior electromagnetic interference (EMI) shielding effectiveness compared to other EMI shielding materials, especially for high electromagnetic (EM) wave absorption. In this study, we investigated the microstructural changes produced by heat treatment and their effect on the EMI shielding properties of Ti-based MXenes. Ti_3_C_2_T_X_ and Ti_2_CT_X_ films were prepared using vacuum filtration and annealed at temperatures up to 300 °C. The microstructures and chemical bonding properties of these heat-treated Ti_3_C_2_T_X_ and Ti_2_CT_X_ films were analyzed, and the EMI shielding effectiveness was measured in the X-band and THz frequency range. The porous Ti_3_C_2_T_X_ film showed higher EM absorption than that calculated using the transfer matrix method. On the other hand, the Ti_2_CT_X_ films had a more densely stacked structure and lower EM absorption. As the heat treatment temperature increased, Ti_3_C_2_T_X_ developed a more porous structure without significant changes in its chemical bonding. Its EM absorption per unit of thickness increased up to 6 dB/μm, while the reflectance remained constant at less than 1 dB/μm after heat treatment. This suggested that the heat treatment of Ti-based MXenes can increase the porosity of the film by removing residual organics without changing the chemical bonds, thereby increasing electromagnetic shielding through absorption.

## 1. Introduction

With the arrival of the fifth-generation network era, 5G technology has been widely applied in a variety of industries, including mobile broadband and autonomous driving, with the goal of improving the quality of communication and service accuracy. However, the popularity of 5G has also increased electromagnetic radiation, which poses potential hazards to electronic devices and human health [1]. The metallic materials (e.g., Ag, Cu, and Ni) that are conventionally used for electromagnetic interference (EMI) shielding suffer from limitations such as a high weight, poor flexibility, poor frequency selectivity, and high cost [2,3,4]. Therefore, there is an urgent need to develop EMI shielding materials that not only demonstrate high shielding effectiveness but are also lightweight, flexible, thermally stable, and cost-effective to promote the sustainable development of related industries [5,6,7,8].

MXenes, a new class of 2D nanomaterials, have received significant attention from the research community owing to their metal-like electrical conductivity and high energy density [9,10,11,12,13,14]. Among the more than 30 MXene materials with different chemical I verify that the sentence retained its intended meaning.compositions synthesized in experiments [13], Ti_3_C_2_T_X_ films exhibit outstanding EMI shielding effectiveness at micro-level thicknesses, superior to that of metal and carbon materials of the same thickness [8,15,16,17,18]. This is attributed to the excellent conductivity and unique layered structure of MXene films.

EMI shielding materials typically prevent the propagation of electromagnetic waves through reflection and absorption. The conductive network in an MXene film contains a large number of free electrons that cause an impedance mismatch when electromagnetic waves are incident on the film, causing them to be reflected [19]. The free-standing Ti_3_C_2_T_X_ film with high electrical conductivity has been reported to exhibit an EMI shielding effectiveness (*SE*) of around 90 dB at a thickness of 45 μm [15]. On the other hand, the charge mismatch of the surface functional groups of MXene generates a dipole polarization loss, while a portion of the electromagnetic waves interact with the induced currents, causing a dielectric loss, thus effectively absorbing the electromagnetic waves [20]. However, since surface reflections still make a large contribution to the electromagnetic shielding performance of Ti_3_C_2_T_X_ films, it is desirable to effectively increase EM absorption by modifying the film’s microstructure without affecting electrical conductivity. The porous structure of MXene films prepared using various methods has been studied to improve EMI *SE* by increasing absorption through multiple internal reflections [21,22,23,24,25,26]. For example, Ti_3_CNT_X_ films with relatively low electrical conductivity have been reported to significantly enhance EMI *SE* through multiple internal reflections at the pore interface within the film [26]. However, there is still a lack of research on the effect of microstructure on the EMI shielding properties of other Ti-based MXenes such as Ti_3_C_2_T_X_ and Ti_2_CT_X_ films, which have excellent electrical conductivity and atmospheric stability.

In this study, Ti-based MXenes, Ti_3_C_2_T_X_ and Ti_2_CT_X_, were fabricated into films with thicknesses of several micrometers and subjected to high-temperature heat treatment. We measured the microstructural changes in and electrical properties of the Ti_3_C_2_T_X_ and Ti_2_CT_X_ films at different heat treatment temperatures. We found that as the heat treatment temperature increased, the interlayer gap decreased to some extent and the porosity in the film increased, owing to the release of adsorbed water molecules and carbon material between the Ti_3_C_2_T_X_ and Ti_2_CT_X_ layers. However, the oxidation state of Ti remained almost unchanged in both Ti_3_C_2_T_X_ and Ti_2_CT_X_ after the heat treatment. In addition, the EMI shielding properties of both Ti_3_C_2_T_X_ and Ti_2_CT_X_ were measured in the GHz frequency range and compared with the results calculated using the transfer matrix method. As a result, the Ti_3_C_2_T_X_ film exhibited excellent EMI shielding effectiveness per thickness of ~7 dB/μm with heat treatment, with the EM reflection remaining unchanged, while the increased absorption contribution arose from its porous internal structure and thin skin depth. We also measured the EMI *SE* of each sample in the THz frequency range using terahertz time-resolved spectroscopy (TDS). The Ti_3_C_2_T_X_ film exhibited a high EMI *SE* of over 60 dB, even in the THz frequency range, demonstrating its potential as an excellent EMI *SE* material for electromagnetic waves over a wide range of frequencies.

## 2. Materials and Methods

### 2.1. Synthesis of Ti-Based MXene and Film Preparation

The Ti-based MXenes, Ti_3_C_2_T_X_ and Ti_2_CT_X_, were synthesized using MAX-phase Ti_3_AlC_2_ and Ti_2_AlC powders (37.5 μm) purchased from Suzhou Bknano Material Co., Ltd. (Suzhou, China). (Figure S1). First, 0.5 g of the MAX-phase Ti_3_AlC_2_ or Ti_2_AlC was slowly added to a mixture of 6 M of HCl (~37 wt% in H_2_O, Sigma-Aldrich, Milwaukee, St. Louis, MO, USA) and 0.5 g of LiF (≥99.0%, AR, Sigma-Aldrich, Milwaukee, America), followed by stirring at 35 °C for 24 h to etch the interlayer Al metal [27,28,29,30]. The resulting solution was transferred to a centrifuge tube, 40 mL of distilled water was added, and the mixture was centrifuged at 3500 rpm for 10 min. After the supernatant was removed, the process was repeated with 40 mL of distilled water until the solution had a pH of 6. Next, the solution was shaken for 20 min, sonicated for 5 min, and centrifuged at 3500 rpm for 10 min. After centrifugation, the supernatant was separated from the sediment to obtain the final dispersions of Ti_3_C_2_T_X_ and Ti_2_CT_X_. The obtained dispersions were vacuum-filtered through Celgard filter paper (pore size: 64 nm) and dried at room temperature for 2 h to produce Ti_3_C_2_T_X_ and Ti_2_CT_X_ films. The prepared Ti_3_C_2_T_X_ and Ti_2_CT_X_ films were heated at a rate of 10 °C/min to 100, 200, or 300 °C in an Ar atmosphere and heat-treated for 6 h. The thickness of the Ti_3_C_2_T_X_ and Ti_2_CT_X_ films was measured using a micrometer, measured at multiple points, and then averaged.

### 2.2. Characterization of MXene Films

The lateral shapes of the Ti_3_C_2_T_X_ and Ti_2_CT_X_ nanoflakes and the cross-sectional structures of the Ti_3_C_2_T_X_ and Ti_2_CT_X_ films were observed using field-emission scanning electron microscopy (FE-SEM, Carl Zeiss, Merlin 3view, The Baden-württemberg Oberkorn, Germany), and the crystal structures were analyzed at room temperature using X-ray diffraction (XRD, Rigaku, Ultima IV, Tokyo, Japan). X-ray photoelectron spectroscopy (XPS, Rigaku, ZSX Primus IV, Tokyo, Japan) was used to examine the changes in the chemical bonding of Ti_3_C_2_T_X_ and Ti_2_CT_X_ materials before and after heat treatment. Ar sputtering was performed prior to the XPS analysis to eliminate the effects of surface adsorbates and oxide layers.

The sheet resistances of the Ti_3_C_2_T_X_ and Ti_2_CT_X_ films were measured using the van der Pauw method with a 4-probe setup, and the electrical conductivities were calculated by dividing the measured sheet resistances by the film thicknesses.

### 2.3. Measurement and Calculation of EMI Shielding Effectiveness of MXene Films

The EMI shielding effectiveness (*SE*) values of the Ti_3_C_2_T_X_ and Ti_2_CT_X_ films were measured using a network analyzer in the X-band frequency range (8.2–12.4 GHz). The EMI *SE* values were then calculated using the S-parameters with the following formulas [31,32]:

$$R={\left\lceil S_{11}\right\rceil }^{2}={\left\lceil S_{22}\right\rceil }^{2}\;T={\left\lceil S_{12}\right\rceil }^{2}={\left\lceil S_{21}\right\rceil }^{2}\;R+A+T=1\;{SE}_{T}={SE}_{R}+{SE}_{A}$$

(1) $${SE}_{T}=10\log\left(\frac{1}{T}\right)=10\log\left(\frac{1}{{\left\lceil S_{12}\right\rceil }^{2}}\right),$$

(2) $${SE}_{R}=10\log\left(\frac{1}{1-R}\right)=10\log\left(\frac{1}{1-{\left|S_{11}\right|}^{2}}\right),$$

(3) $${SE}_{A}=10\log\left(\frac{1-R}{T}\right)=10\log\left(\frac{{1-\left|S_{11}\right|}^{2}}{{\left|S_{12}\right|}^{2}}\right),$$

where *R*, *A*, and *T* are the reflection, absorption, and transmission coefficients, respectively. *SE_R_* and *SE_A_* represent the reflection and absorption *SE* values, respectively, and *SE_T_* is the total *SE*. *SE_A_* implicitly includes the effectiveness from multiple internal reflections, *SE_M_*.

The EMI *SE* of the Ti_3_C_2_T_X_ and Ti_2_CT_X_ films at 0.5–2.0 THz was measured using a conventional terahertz time-domain spectrometer (TDS) set up with two pairs of photoconductive antennas [18,33]. The transmission through a bare substrate (undoped high-resistance silicon) was measured as a reference signal, after which Ti_3_C_2_T_X_ and Ti_2_CT_X_ films were attached to the substrate and the sample signal was recorded. The normalized transmission (EMI *SE*) was calculated using the following formula [34]:

$$EMI\;SE(dB)=-20log(\frac{sample\;signal}{reference\;signal}).$$

This formula was used to determine the EMI *SE* values of the Ti_3_C_2_T_X_ and Ti_2_CT_X_ films.

The transfer matrix method was employed to calculate the EMI *SE_T_*, *SE_A_*, and *SE_R_* values of the Ti_3_C_2_T_X_ and Ti_2_CT_X_ films [31,35]. Here, we assumed a normally incident plane wave for Ti_3_C_2_T_X_ and Ti_2_CT_X_ films in air. The complex refractive index of the Ti_3_C_2_T_X_ and Ti_2_CT_X_ films was set to $n=\sqrt{\sigma /2\omega \epsilon_{0}}\left(1+i\right)$ because the Ti_3_C_2_T_X_ and Ti_2_CT_X_ films were conductive with high electrical conductivity, where σ is the conductivity of the Ti_3_C_2_T_X_ or Ti_2_CT_X_ film, ω is the angular frequency, and ε_0_ is the vacuum permittivity. The wavenumber values in vacuum (*k*_0_) and in the Ti_3_C_2_T_X_ or Ti_2_CT_X_ film (*k*) were *k*_0_ = *ω*⁄*c* and *k* = *nω*⁄*c* = *k*_1_ + *ik*_2_, respectively, where c is the speed of light in vacuum. The reflection and transmission coefficients (*R* and *T*, respectively) for the TE mode were then calculated using the transfer matrix:

$$T={\left|2k_{0}/\left(k_{0}+k_{1}\right)\right|}^{2}exp\left(-2k_{2}h\right)\text{,}\;R={\left|\left(k_{0}-k_{1}\right)/\left(k_{0}+k_{1}\right)\right|}^{2}\text{,}$$

where *h* is the thickness of the Ti_3_C_2_T_X_ or Ti_2_CT_X_ film. Then, *SE_T_*, *SE_R_*, and *SE_A_* were calculated using Equations (1), (2), and (3), respectively [31].

## 3. Results and Discussion

### 3.1. Synthesis of Ti_3_C_2_T_X_ and Ti_2_CT_X_ Nanosheets

**Figure 1:**
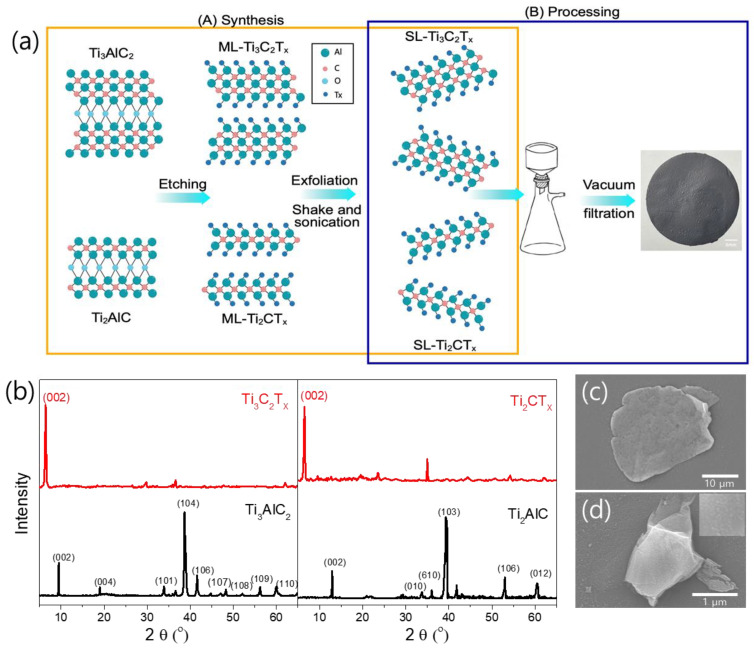
(**a**) Schematic showing the synthesis process of Ti-based MXene; (**b**) XRD spectra of Ti_3_C_2_T_X_ and Ti_2_CT_X_, compared with the MAX phases of Ti_3_AlC_2_ and Ti_2_AlC, respectively. SEM images of the synthesized (**c**) Ti_3_C_2_T_X_ and (**d**) Ti_2_CT_X_ flakes, where the inset in (**d**) shows a magnified SEM image of Ti_2_CT_X_ with nanoparticles on the surface.

The process of synthesizing Ti_3_C_2_T_X_ and Ti_2_CT_X_, and preparing the films, is illustrated in Figure 1a. XRD measurements were used to confirm the synthesis of Ti_3_C_2_T_X_ and Ti_2_CT_X_ via MAX-phase etching. As shown in the XRD spectra in Figure 1b, the main peaks of Ti_3_AlC_2_ and Ti_2_AlC did not appear in the XRD patterns of Ti_3_C_2_T_X_ and Ti_2_CT_X_, which indicated the complete conversion of MAX to MXene phases. The (002) characteristic peaks of Ti_3_C_2_T_X_ and Ti_2_CT_X_ were observed at 6.43° and 6.50°, corresponding to interlayer spacing values of 15.77 and 13.56 Å, respectively. The SEM images in Figure 1c,d show the synthesized Ti_3_C_2_T_X_ and Ti_2_CT_X_ nanosheets, respectively, which had measured lateral sizes of approximately 30 and 2.5 μm, respectively (Figure S2). The large difference in the lateral sizes of the Ti_3_C_2_T_X_ and Ti_2_CT_X_ nanosheets was due to the easier formation of TiO_2_ particles on Ti_2_CT_X_, which causes fractures throughout the nanosheet [36,37,38,39]. In fact, as shown in the SEM image in Figure 1d, the surfaces of the Ti_2_CT_X_ nanosheets contained many small particles that appeared to be TiO_2_.

### 3.2. Structural Changes in Ti_3_C_2_T_X_ and Ti_2_CT_X_ Films Produced by Heat Treatment

**Figure 2:**
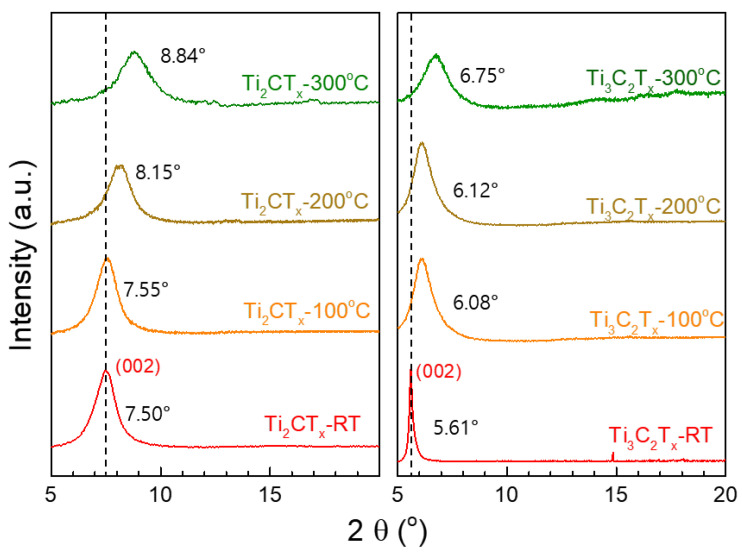
XRD spectra of Ti_3_C_2_T_X_ and Ti_2_CT_X_ heat-treated at different temperatures.

Micrometer-thick Ti_3_C_2_T_X_ and Ti_2_CT_X_ films prepared by vacuum filtration were heat-treated at various temperatures. XRD and SEM analyses were performed to observe the microstructural changes in the Ti_3_C_2_T_X_ and Ti_2_CT_X_ films. The XRD spectra in Figure 2 show the changes in the d-spacing of (002) with the heat treatment temperature, demonstrating that the d-spacing of (002) of the Ti_3_C_2_T_X_ and Ti_2_CT_X_ films gradually decreased with increasing heat treatment temperature, as the (002) peak position shifted to the right. Table 1 lists the (002) d-spacing of the Ti_3_C_2_T_X_ and Ti_2_CT_X_ films calculated by Bragg’s law, $n\lambda =2d_{002}\sin\theta \;(\lambda =1.54\;Å)$, using the peak position in Figure 2. The d-spacing of (002) of the as-prepared Ti_3_C_2_T_X_ film (15.75 Å) was larger than that of Ti_2_CT_X_ (11.78 Å). However, the d-spacing of (002) for the two films decreased to 13.08 Å and 10.00 Å after heat treatment at 300 °C, respectively, showing similar reductions of approximately 17% and 15%, respectively.

**Figure 3:**
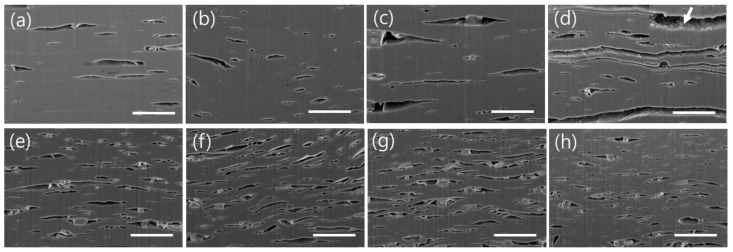
Cross-sectional SEM images of as-prepared (**a**) Ti_3_C_2_T_X_, (**e**) Ti_2_CT_X_ films, (**b**–**d**) Ti_3_C_2_T_X_ and (**f**–**h**) Ti_2_CT_X_ films heat-treated at 100, 200, and 300 °C, respectively (from left to right). The scale bar is 1 μm.

Figure 3a–h show cross-sectional SEM images of the Ti_3_C_2_TX and Ti_2_CT_X_ films before heat treatment and after heat treatment at 100, 200, and 300 °C, showing changes in the layered structure according to the heat treatment temperature. As shown in Figure 3a,e, the as-prepared Ti_3_C_2_T_X_ and Ti_2_CT_X_ films had a well-stacked layered structure of nanoflakes, but there were numerous voids of various sizes within them. In particular, Ti_3_C_2_T_X_ contained a larger number of smaller voids. This was likely because Ti_2_CT_X_, which had a lateral size of 2 μm, formed a more densely packed layered structure compared to Ti_3_C_2_T_X_, which had a lateral size of approximately 30 µm, as shown in the SEM image in Figure 1c. After heat treatment, for both the Ti_3_C_2_T_X_ and Ti_2_CT_X_ films, the number of voids increased and the spacing of the interlayer gaps formed by the connected voids gradually widened with the heat treatment temperature, as shown in Figure 3a–h. The increase in voids with the heat treatment temperature was likely due to the formation of internal empty spaces resulting from the decrease in the interlayer spacing values of Ti_3_C_2_T_X_ and Ti_2_CT_X_, as indicated by the XRD spectra in Figure 2. As shown in Figure 3a–h, the widening of the interlayer gaps owing to heat treatment was more pronounced in Ti_2_CT_X_. This was because Ti_2_CT_X_ was easily oxidized when exposed to the external environment in the void regions, generating TiO_2_ on the surface, which further weakened the interlayer bonds and led to interlayer separation (inset of Figure 3d). In fact, large particles are occasionally observed within the film, as confirmed by the XRD spectra in Figure S3, which are presumed to be LiF particles that were not cleaned during the etching process. However, the amount of LiF is very small, as shown in the XRD spectra, so it is believed that the LiF impurities in the film do not affect the film’s properties.

### 3.3. Electrical Properties of Ti_3_C_2_T_x_ and Ti_2_CT_x_ After Heat Treatment

**Figure 4:**
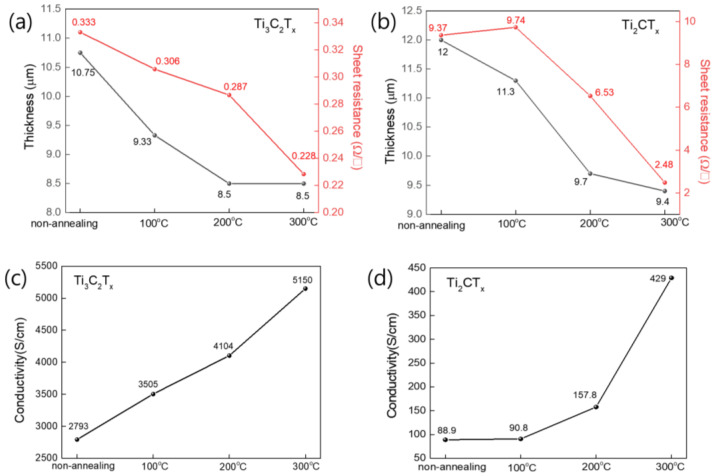
(**a**,**b**) Plots of thickness and sheet resistance values of Ti_3_C_2_T_X_ and Ti_2_CT_X_ films heat-treated at different temperatures, respectively. (**c**,**d**) Conductivities of Ti_3_C_2_T_X_ and Ti_2_CT_X_ films calculated using the thickness and sheet resistivity values in (**a**) and (**b**), respectively.

Figure 4a,b show the changes in the thickness and sheet resistance of the Ti_3_C_2_T_x_ and Ti_2_CT_X_ films after heat treatment at different temperatures. These values were used to calculate the conductivities of the Ti_3_C_2_T_X_ and Ti_2_CT_X_ films, as shown in Figure 4c and Figure 4d, respectively. The thicknesses of the Ti_3_C_2_T_X_ and Ti_2_CT_X_ films decreased gradually as the heat treatment temperature increased to 200 °C, with little change after heat treatment at 300 °C. In contrast, the sheet resistances of the Ti_3_C_2_T_X_ and Ti_2_CT_X_ films decreased gradually as the heat treatment temperature increased, as shown in Figure 4b. In particular, the change in the sheet resistance of Ti_2_CT_X_ with heat treatment was very large, with the sheet resistance decreasing by more than 70% from 9.37 to 2.48 Ω/sq after heat treatment at 300 °C. The sheet resistance of the Ti_3_C_2_T_X_ film decreased from 0.33 to 0.23 Ω/sq after heat treatment at 300 °C. The conductivities of the Ti_3_C_2_T_X_ and Ti_2_CT_X_ films, which were calculated by multiplying the thickness by the sheet resistance, increased with the heat treatment temperature, as shown in Figure 4c. Overall, the conductivity of the Ti_3_C_2_T_X_ films was significantly higher than that of the Ti_2_CT_X_ films; however, the conductivity of Ti_2_CT_X_ increased more rapidly than that of Ti_3_C_2_T_X_. The electrical conductivity of Ti_3_C_2_T_X_ showed an increase of approximately 1.8 times, rising from 2793 S/m before heat treatment to 5150 S/cm after heat treatment at 300 °C. In contrast, Ti_2_CT_X_ showed a significant increase in electrical conductivity of 4.8 times after heat treatment, from 88.9 S/cm before heat treatment to 429 S/cm after heat treatment at 300 °C. The difference in increases in the conductivities of the Ti_3_C_2_T_X_ and Ti_2_CT_X_ films with heat treatment could be attributed to the size difference between the Ti_3_C_2_T_X_ and Ti_2_CT_X_ flakes. According to the percolation theory, as the size of the flakes decreased, the increase in the contribution of interlayer transport, which had a relatively higher resistance, led to an increase in the overall resistance [40]. Moreover, the removal of the moisture and organic materials present between the layers by the heat treatment facilitated interlayer charge transport and significantly increased the conductivity [41]. Therefore, it is believed that the smaller flakes of Ti_2_CT_X_ experienced a greater reduction in the resistance between flakes owing to heat treatment, resulting in a more significant change in the overall electrical conductivity.

### 3.4. Compositional Changes in Ti_3_C_2_T_X_ and Ti_2_CT_X_ Films Produced by Heat Treatment

**Figure 5:**
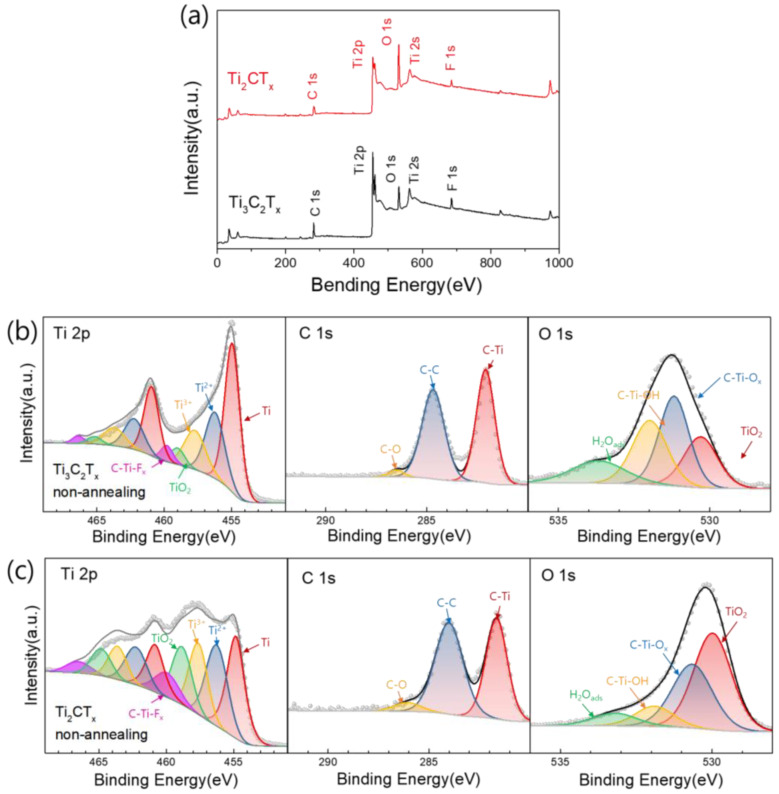
(**a**) XPS survey spectra of Ti_3_C_2_T_X_ and Ti_2_CT_X_. (**b**,**c**) High-resolution XPS spectra scanned over Ti 2p, C 1s, and O 1s of Ti_3_C_2_T_X_ and Ti_2_CT_X_ films, respectively, before heat treatment.

XPS analyses were performed to obtain information on the changes in the surface chemistry of Ti_3_C_2_T_X_ and Ti_2_CT_X_ films upon heat treatment (Figure S4). First, we compared the atomic compositions of the as-prepared Ti_3_C_2_T_X_ and Ti_2_CT_X_ from the survey spectra, and the chemical bonding of their main elements from the high-resolution XPS spectra, as shown in Figure 5a–c. Figure 5a is an XPS survey spectrum showing the composition of Ti, C, O, and F, the main elements of Ti_3_C_2_T_X_ and Ti_2_CT_X_. It shows that Ti_3_C_2_T_X_ had a higher number of Ti and C atoms than Ti_2_CT_X_ but a lower number of O atoms. Figure 5b,c show the Ti 2p, C 1s, and O 1s XPS profiles of Ti_3_C_2_T_X_ and Ti_2_CT_X_ before annealing, respectively. The component fitting of the XPS peaks of Ti_3_C_2_T_X_ and Ti_2_CT_X_ was performed following the method outlined by Halim et al. [42]. The Ti 2p spectrum of Ti_3_C_2_T_X_ in Figure 5b shows that the Ti peak attributed to C–Ti–O(OH) was the most dominant, and the proportion of peaks corresponding to the higher oxidation states of Ti, such as Ti^2+^, Ti^3+^, and Ti^4+^ (TiO_2_), gradually decreased. In contrast, as seen in Figure 5c, Ti_2_CT_X_ had a significantly larger proportion of higher oxidation states, such as Ti^2+^ and Ti^3+^, with the Ti^4+^ peak corresponding to TiO_2_ becoming particularly prominent. These results were also observed in the O 1s spectra, where Ti_3_C_2_T_X_ primarily showed C–Ti–OH and C–Ti–O_X_, whereas Ti_2_CT_X_ had TiO_2_ as its most significant component. Meanwhile, the C 1s spectra of both Ti_3_C_2_T_X_ and Ti_2_CT_X_ exhibited a Ti–C peak at ~282 eV, corresponding to the carbon bonds located in the Ti octahedra, and a graphite-like carbon C=C peak at ~284.5 eV, originating from organic materials mainly produced during MAX etching, with both peaks occupying similar proportions in each case [43].

**Figure 6:**
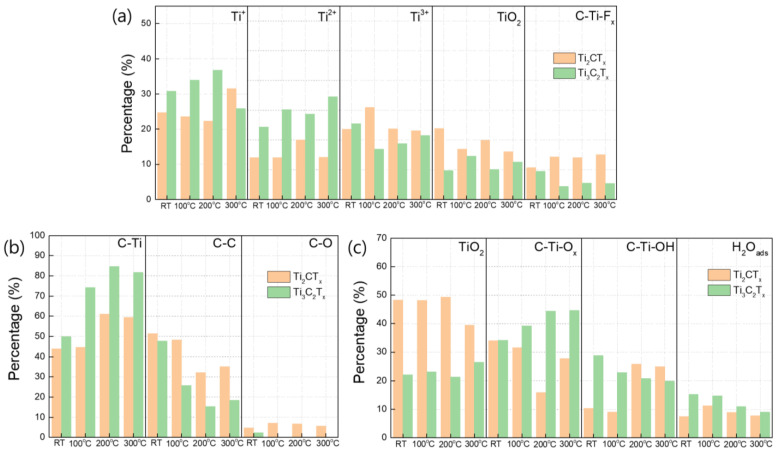
Histograms showing the percentage changes in the (**a**) Ti, (**b**) C, and (**c**) O bonding states in the Ti_3_C_2_T_X_ and Ti_2_CT_X_ films as the heat treatment temperature increased.

Figure 6a illustrates the changes in the Ti bonding state with the various heat treatment temperatures derived from the Ti 2p spectra of Ti_3_C_2_T_X_ and Ti_2_CT_X_ (Figure S5). First, as previously mentioned for Figure 5b,c, Ti_3_C_2_T_X_ contained a larger proportion of low-oxidation-state Ti bonds, specifically Ti and Ti^2+^, whereas Ti_2_CT_X_ had a greater proportion of Ti bonds in higher oxidation states, such as Ti^3+^ and Ti^4+^. A comparison of the changes in the Ti bonding states with the heat treatment temperature shows that the proportion of low-oxidation-state Ti and Ti^2+^ bonds in Ti_3_C_2_T_X_ tended to increase, while the proportion of high-oxidation-state Ti^3+^ and Ti^4+^ bonds in Ti_2_CT_X_ tended to decrease, as the heat treatment temperature increased, although there were slight deviations in the Ti bonding state of Ti_3_C_2_T_X_ at 300 °C. The decrease in the proportion of C–C bonds with heat treatment, as shown in Figure 6b, indicates the removal of the organic materials present in Ti_3_C_2_T_X_ and Ti_2_CT_X_ with an increase in the heat treatment temperature (Figure S5). Remarkably, the removal of organic materials via heat treatment was more pronounced for Ti_3_C_2_T_X_ than for Ti_2_CT_X_. It is also noteworthy that the C–O bonds, which are known to arise from the solvents used in the synthesis process, almost disappeared in Ti_3_C_2_T_X_ after heat treatment, whereas in Ti_2_CT_X_, they still accounted for more than 5% and showed little change with heat treatment. An examination of the changes in the O bonding states obtained from the O 1s spectra at different heat treatment temperatures, as shown in Figure 5c, indicates that as the heat treatment temperature increased, Ti_3_C_2_T_X_ showed a slight increase in the proportion of TiO_2_ and C–Ti–O bonds, whereas C–Ti–OH and H_2_O_ads_ tended to gradually decrease (Figure S5). Ti_2_CT_X_, on the other hand, showed little change in the proportion of TiO_2_, except after heat treatment at 300 °C, but had a much higher proportion than Ti_3_C_2_T_X_. It is also noteworthy that the proportion of C–Ti–OH bonds increased and that H_2_O_ads_ showed little change as the heat treatment temperature increased. Based on the analysis of the Ti and O bonding states, it can be concluded that both Ti_3_C_2_T_X_ and Ti_2_CT_X_ exhibited a decrease in the oxidation states of the Ti bonds owing to heat treatment. However, Ti_3_C_2_T_X_, which predominantly featured low-oxidation-state Ti bonds, primarily showed an increase in the proportions of Ti and Ti^2+^ bonds, whereas Ti_2_CT_X_, which was characterized by higher-oxidation-state Ti bonds, mainly displayed a decrease in the proportions of Ti^3+^ and Ti^4+^ bonds. Additionally, organic materials, residual solvents, and moisture adsorbed on the surface were effectively removed from Ti_3_C_2_T_X_ by heat treatment, whereas they were less effectively removed from Ti_2_CT_X_. This difference could be attributed to the fact that, as shown in the SEM images in Figure 3a–h, Ti_2_CT_X_ had a very densely stacked layered structure, whereas Ti_3_C_2_T_X_ had a relatively porous layered structure that facilitated the removal of organic materials, moisture, and solvents.

### 3.5. EMI Shielding Properties of Ti_3_C_2_T_X_ and Ti_2_CT_X_ Films in High-Frequency Range

**Figure 7:**
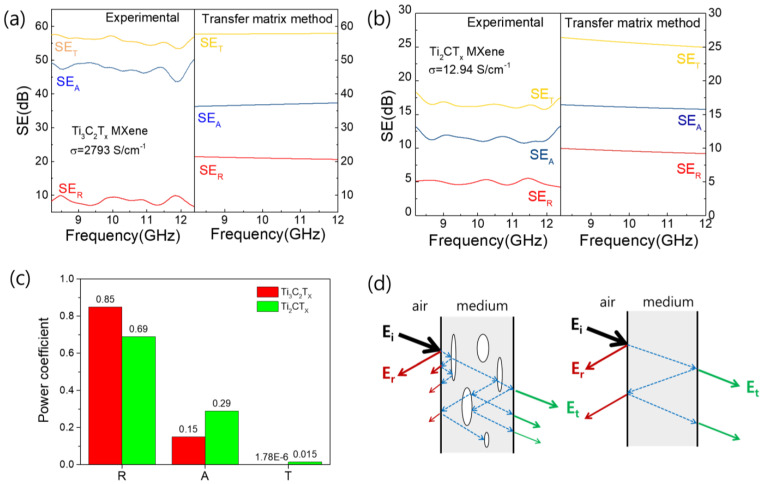
(**a**,**b**) Measured EMI *SE_T_*, *SE_A_*, and *SE_R_* values for Ti_3_C_2_T_X_ and Ti_2_CT_X_ films, compared to values calculated using transfer matrix method. (**c**) Power coefficients, *R*, *A*, and *T*, of Ti_3_C_2_T_X_ and Ti_2_CT_X_ films. (**d**) Schematic illustrating the multiple reflections of electromagnetic waves with and without pores inside a material.

Figure 7a,b show the measured EMI *SE* values of the as-prepared Ti_3_C_2_T_X_ and Ti_2_CT_X_ in the X-band frequency range (8.2–12.4 GHz) in comparison with the values calculated using the transfer matrix method [31]. The electrical conductivities of the Ti_3_C_2_T_X_ and Ti_2_CT_X_ films used in this experiment were 2793 and 12.94 S/cm, respectively, and their thicknesses were 10.75 and 12.05 μm, respectively. The skin depths (*δ*) of Ti_3_C_2_T_X_ and Ti_2_CT_X_ were calculated to be 10.55 and 59.09 μm at 8.2 GHz, respectively, based on the following formula: $\delta =\sqrt{1/\pi \sigma \mu f}$, where σ, *μ*, and *f* are the conductivity, permeability, and frequency, respectively. Because the Ti_2_CT_X_ film thickness of 12.05 μm was much thinner than the skin depth of 59.09 μm, we calculated EMI *SE* using the transfer matrix method rather than the Simon equation to include multiple reflections [30]. Figure 7a shows that the measured total EMI *SE* (*SE_T_*) of Ti_3_C_2_T_X_ was very close to the calculated value obtained using the transfer matrix method. However, the measured reflected EMI *SE* (*SE_R_*) was smaller than the calculated value, while the absorbed EMI *SE* (*SE_A_*) was the opposite, with the measured value being larger than the calculated value. In contrast, Figure 7b shows that all the measured EMI *SE* (*SE_T_*, *SE_A_*, *SE_R_*) values of Ti_2_CT_X_ were lower than the values calculated using the transfer matrix method. These results were likely due to the fact that Ti_2_CT_X_ was easily oxidized in the atmosphere, resulting in a decrease in electrical conductivity over time. In practice, electromagnetic shielding is performed a few days after sample fabrication, and the electrical conductivity of Ti_2_CT_X_ films typically decreases by 3% after 4 days (Figure S6) [37]. Notably, in Ti_2_CT_X_, the contributions of E_A_ and E_R_ to E_tot_ were not significantly different between the experiments and calculations, unlike in Ti_3_C_2_T_X_. The differences appeared to be attributable to differences in the internal structures of the stacked films of Ti_3_C_2_T_X_ and Ti_2_CT_X_. As previously discussed for Figure 3a,e, Ti_3_C_2_T_X_ had a porous structure with many voids, whereas Ti_2_CT_X_ had a relatively densely stacked structure. In order to further clarify the EMI shielding mechanism, the power coefficients of the Ti_3_C_2_T_X_ and Ti_2_CT_X_ films were calculated using Equations (1)–(3) [44]. As shown in Figure 7c, the near-zero T value of Ti_3_C_2_T_X_ indicates that the incident electromagnetic waves hardly penetrate the Ti_3_C_2_T_X_ film, and the reflection is still the dominant shielding mechanism in Ti_3_C_2_T_X_ since R is higher than A. The Ti_2_CT_X_ film with a T of 0.015 showed a higher A than Ti_3_C_2_T_X_, which indicates that the Ti_2_CT_X_ film is unfavorable for the blocking of EM waves but has a higher absorption contribution to shielding than Ti_3_C_2_T_X_. This is not due to increased electromagnetic absorption in Ti_2_CT_X_, but rather to the low electrical conductivity of Ti_2_CT_X_, which results in less electromagnetic reflection from the surface and thus more absorption contributing to shielding. The many small TiO_2_ particles observed in the SEM image in Figure 3 could contribute to the increased electromagnetic absorption of the Ti_2_CT_X_ film due to dielectric losses, but conversely, the oxidation of Ti_2_CT_X_ could also result in decreased absorption due to decreased electrical conductivity. On the other hand, the absorption contribution to the shielding of Ti_3_C_2_T_X_, defined as the ratio of *A* to (*A* + *R*), is 0.15, which is much larger than the expected value of 0.007 from the transfer matrix method (Figure S7). This means that the absorption contribution is increased by the internal pores in the Ti_3_C_2_T_X_ film, as already discussed in Figure 7a,b. The schematics in Figure 7d illustrate the multiple reflections that can occur in materials with and without pores in the interior; in porous materials, more absorption can occur inside the material by reflections from the surface of the pores. Therefore, Ti_3_C_2_T_X_, which had more internal pores, was considered to have a larger *SE_A_* than that calculated using the transfer matrix method.

**Figure 8:**
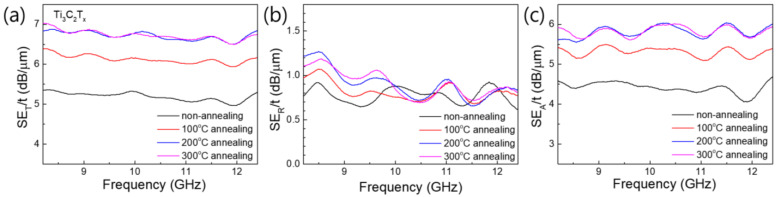
EMI *SE* values normalized to thickness: (**a**) *SE_T_*/*t*, (**b**) *SE_R_*/*t*, and (**c**) *SE_A_*/*t* of Ti_3_C_2_T_X_ films heat-treated at different temperatures.

Figure 8a–c show the *SE_T_*, *SE_A_*, and *SE_R_* values normalized to the thickness of the as-prepared Ti_3_C_2_T_X_ and Ti_3_C_2_T_X_ films heat-treated at 100, 200, and 300 °C, respectively. The *SE_T_* of Ti_3_C_2_T_X_ normalized to thickness (*SE_T_*/*t*) increased with the heat treatment temperature up to 200 °C, with no significant increase after heat treatment at 300 °C. Interestingly, the *SE_R_* normalized to thickness (*SE_R_*/*t*) was almost the same for all the Ti_3_C_2_T_X_ films heat-treated at different temperatures, despite the difference in their electrical conductivities. As previously discussed in the XPS analysis of the Ti_3_C_2_T_X_ films heat-treated at different temperatures, the increase in the conductivity of Ti_3_C_2_T_X_ by heat treatment was mainly caused by the removal of organic material or adsorbed solvents between the Ti_3_C_2_T_X_ flakes and not by chemical bonding or compositional changes in Ti_3_C_2_T_X_. Therefore, because the electrical conductivity of the Ti_3_C_2_T_X_ flake was hardly changed by heat treatment, *SE_R_*, which was mainly caused by electromagnetic waves reflected from the surface of the Ti_3_C_2_T_X_ film, did not seem to change significantly after heat treatment. In contrast, the *SE_A_* normalized to thickness (*SE_A_*/*t*) increased with the heat treatment temperature, similarly to *SE*_T_/*t*. These results can be explained by two factors: the electrical conductivity and the porous structure. First, a sample with a higher electrical conductivity, that is, less skin depth, absorbed electromagnetic waves better for the same thickness; therefore, when the Ti_3_C_2_T_X_ was heat-treated at higher temperatures, it had a larger *SE_A_*/*t*. Second, as previously discussed, the Ti_3_C_2_T_X_ film heat-treated at higher temperatures had a more porous structure with many voids, which allowed it to absorb electromagnetic waves more efficiently through multiple reflections. In conclusion, the increase in the *SE_T_*/*t* value of Ti_3_C_2_T_X_ produced by heat treatment was mainly caused by the increase in absorption due to changes in the stacking structure of the Ti_3_C_2_T_X_ flakes, not by changes in the Ti_3_C_2_T_X_ material itself.

In fact, there have been many efforts to improve EMI shielding by fabricating composites or foams to introduce interfaces for internal scattering [45,46,47,48]. However, adding conductive nanowires or magnetic particles to the MXene film changes the electrical conductivity of the EMI shielding material, which in turn affects the reflection and absorption of EMI. Lightweight MXene foam, fabricated from MXene film through a foaming process, exhibited a significant increase in EMI *SE* for the same mass, but with a very large increase in thickness. In comparison, the porous MXene film prepared by a simple heat treatment method in this study exhibited an EMI *SE* of about 60 dB at a film thickness of about 9 μm, which was superior to MXene foam with an EMI *SE* of about 40 dB at the same thickness.

**Figure 9:**
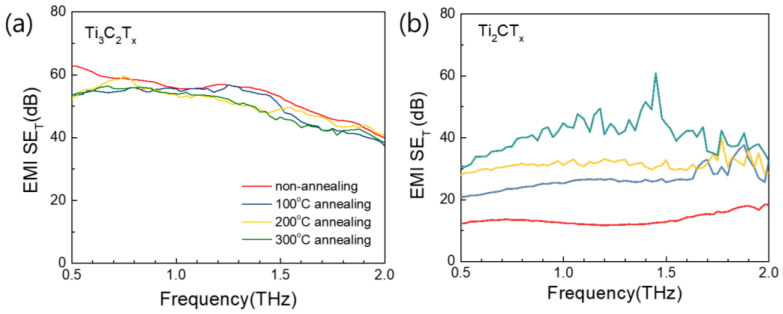
Total EMI *SE* values of (**a**) Ti_3_C_2_T_X_ and (**b**) Ti_2_CT_X_ films heat-treated at different temperatures in THz frequency range.

We also measured the electromagnetic shielding of Ti_3_C_2_T_X_ and Ti_2_CT_X_ films heat-treated at different temperatures in the THz region [33,49]. Although the difference between the Ti_3_C_2_T_X_ films heat-treated at different temperatures could not be measured due to equipment limitations, Figure 9 shows that the electromagnetic shielding of all the Ti_3_C_2_T_X_ samples in the 0.5–2.0 THz range was higher than that of Ti_2_CT_X_. Therefore, Ti-based MXenes, including Ti_3_C_2_T_X_ and Ti_2_CT_X_, have high EMI *SE*, with values generally greater than 20 dB at frequencies higher than the GHz range, which satisfy industry application requirements for 5G technology.

## 4. Conclusions

In summary, we studied the EMI effectiveness of heat-treated Ti_3_C_2_T_X_ and Ti_2_CT_X_ films in the X-band frequency range and analyzed the effects of microstructural changes in the Ti_3_C_2_T_X_ and Ti_2_CT_X_ films on electromagnetic wave reflection and absorption. The Ti_3_C_2_T_X_ film was composed of large flakes with a lateral size of tens of micrometers and had a relatively porous structure. Conversely, the Ti_2_CT_X_ film, which was composed of flakes with a measured size of a few micrometers, demonstrated a more densely packed structure with minimal internal pores. After heat-treating the Ti_3_C_2_T_X_ and Ti_2_CT_X_ films at 300 °C, an analysis of the changes in their microstructures and chemical bonding revealed that the oxidation state of Ti decreased in both films; however, no significant changes in the chemical bonding of the materials themselves were observed. Nonetheless, the reduction in organic content within the Ti_3_C_2_T_X_ film was more pronounced, which is believed to be due to the more porous internal structure of Ti_3_C_2_T_X_. In conclusion, the EMI electromagnetic wave absorption effectiveness of Ti_3_C_2_T_X_ was not only higher than predicted by the transfer matrix method, but it also gradually increased with heat treatment, showing a value of approximately 6 dB per micrometer of thickness after heat treatment at 300 °C, while the electromagnetic wave reflectance remained almost unchanged. Additionally, Ti_3_C_2_T_X_ exhibited a high EMI *SE* of >60 dB in the THz frequency range. Therefore, we anticipate that Ti_3_C_2_T_X_ films made from large flakes with sizes of tens of micrometers and subjected to high-temperature heat treatment will be useful as an electromagnetic wave-shielding material, particularly for absorption across a wide range of high frequencies.

## Figures and Tables

**Table 1:** The d-spacing of (002) of Ti_3_C_2_T_X_ and Ti_2_CT_X_ heat-treated at different temperatures.

Heat Treatment Temperature	Ti_3_C_2_T_X_	Ti_2_CT_X_
w/o	11.78 Å	15.75 Å
100 °C	11.71 Å	14.52 Å
200 °C	10.85 Å	14.44 Å
300 °C	10.00 Å	13.08 Å

## Data Availability

The data presented in this study are available upon request from the corresponding author.

## Supplementary Materials

The following supporting information can be downloaded at: https://www.mdpi.com/article/10.3390/nano15090676/s1, Figure S1. SEM images of (a–c) Ti_3_C_2_T_X_ and (d–f) Ti_2_CT_X_ flakes, comparing their lateral sizes. Figure S2. SEM images of (a) Ti_2_AlC and (b) Ti_3_AlC_2_ MAX powder. Figure S3. SEM images of (a) Ti_2_AlC and (b) Ti_3_AlC_2_ MAX powder. Figure S4. Compositional changes in the main elements (Ti, C, O, F and Cl) of Ti_3_C_2_T_X_ and Ti_2_CT_X_ upon heat treatment at different temperatures. Figure S5. High resolution XPS spectra of (a,b) Ti 2p, (c,d) C 1s, and (e,f) O 1s of Ti_3_C_2_T_X_ and Ti_2_CT_X_ upon heat treatment. Figure S6 Changes in the electrical conductivity of Ti_3_C_2_T_X_ and Ti_2_CT_X_ film over time when exposed to the atmosphere. Figure S7. (a,b) Power coefficient, R, A and T, of Ti_3_C_2_T_X_ and Ti_2_CT_X_ film calculated with *SE_R_*, *SE_A_* and *SE_T_* measured experimentally and calculated by transfer matrix method, respectively, using Equations (1)–(3).
