# Supplementary material for: The Effect of Microstructural Changes Produced by Heat Treatment on the Electromagnetic Interference Shielding Properties of Ti-Based MXenes
Source: Nanomaterials (Basel). 2025 Apr 29;15(9):676. doi: 10.3390/nano15090676 (PMC12073403; doi:10.3390/nano15090676)
Supplement: Supplementary file 1 [file nanomaterials-15-00676-s001.zip › nanomaterials-3574814-supplementary.pdf]

Supporting Information

The Effect of Microstructural Changes Produced by Heat Treatment on the Electromagnetic Interference Shielding Properties of Ti-Based MXenes

Xue Han [1], Jae Jeong Lee [1], Ji Soo Kyoung [2] and Yun Sung Woo [1,*]

[1] Department of Materials Science and Engineering, Dankook University, Dandae-ro 119, Dongnam-gu, Cheonan-si 31116, Chungnam-do, Republic of Korea; axh1999a@dankook.ac.kr (X.H.); jaejeong.lee@dankook.ac.kr (J.J.L.)

[2] Department of Physics, Dankook University, Dandae-ro 119, Dongnam-gu, Cheonan-si 31116, Chungnam-do, Republic of Korea; kyoungjs@dankook.ac.kr

* Correspondence: yunswoo@dankook.ac.kr

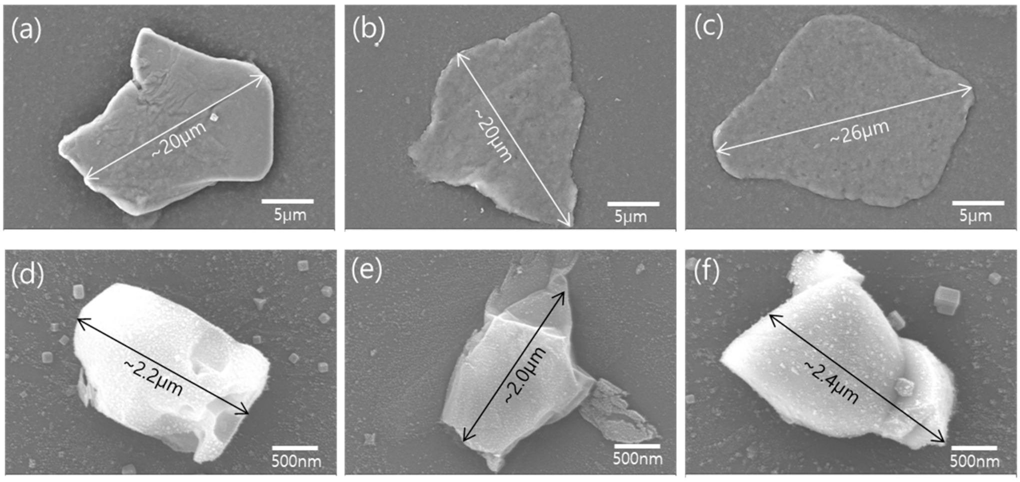

Fig S1. SEM images of (a)-(c) $Ti_3C_2T_X$ and (d)-(f) Ti_2CT_X flakes, comparing their lateral sizes.

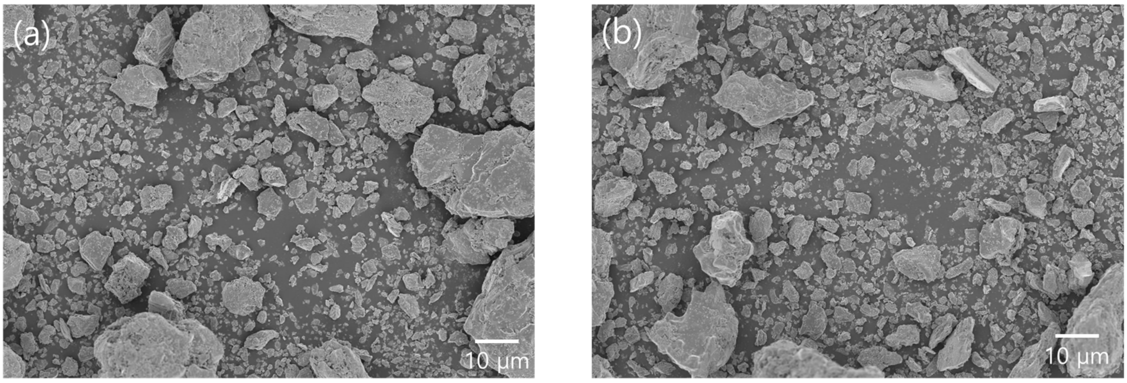

Fig S2. SEM images of (a) Ti_2AlC and (b) Ti_3AlC_2 MAX powder

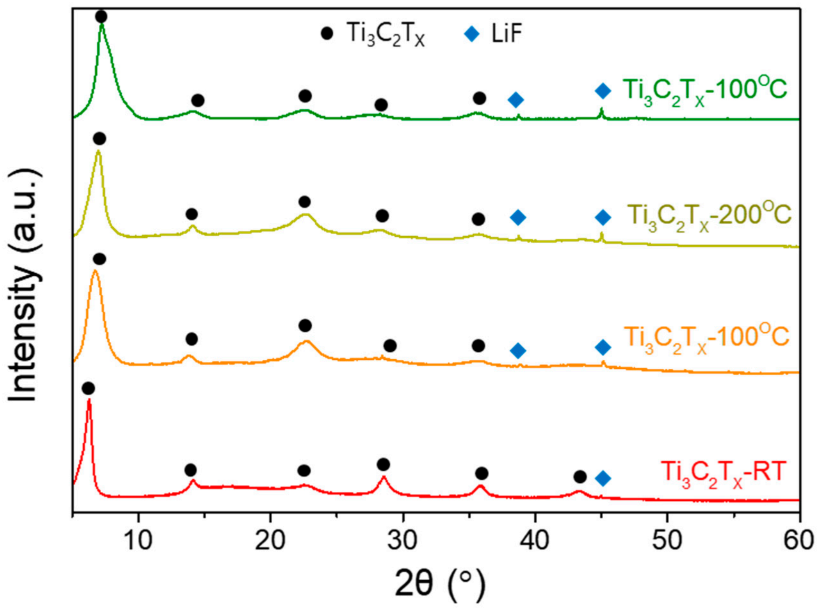

Fig S3. SEM images of (a) Ti_2AlC and (b) Ti_3AlC_2 MAX powder

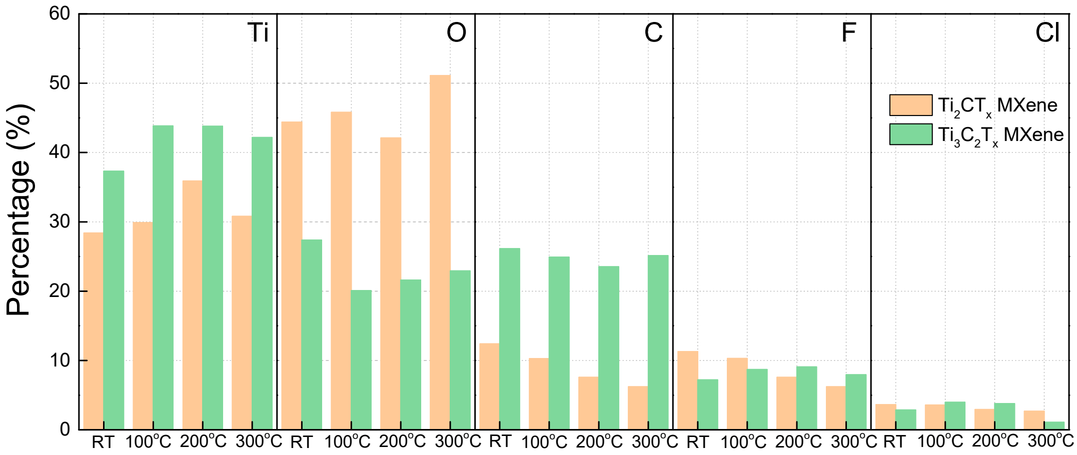

Fig. S4 Compositional changes in the main elements (Ti, C, O, F and Cl) of $Ti_3C_2T_X$ and Ti_2CT_X upon heat treatment at different temperatures.

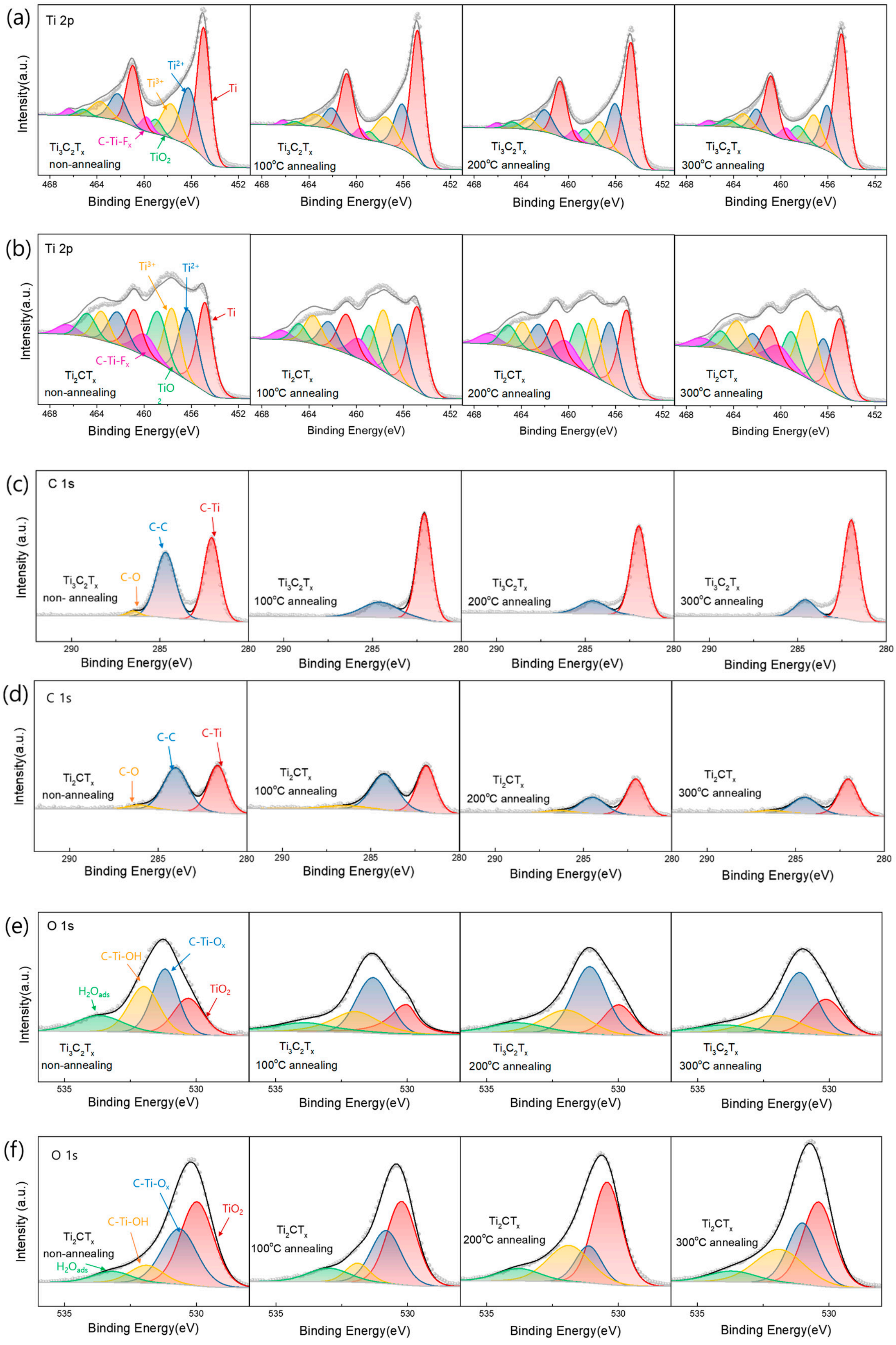

Fig. S5 High resolution XPS spectra of (a), (b) Ti 2p, (c), (d) C 1s, and (e), (f) O 1s of $Ti_3C_2T_X$ and Ti_2CT_X upon heat treatment.

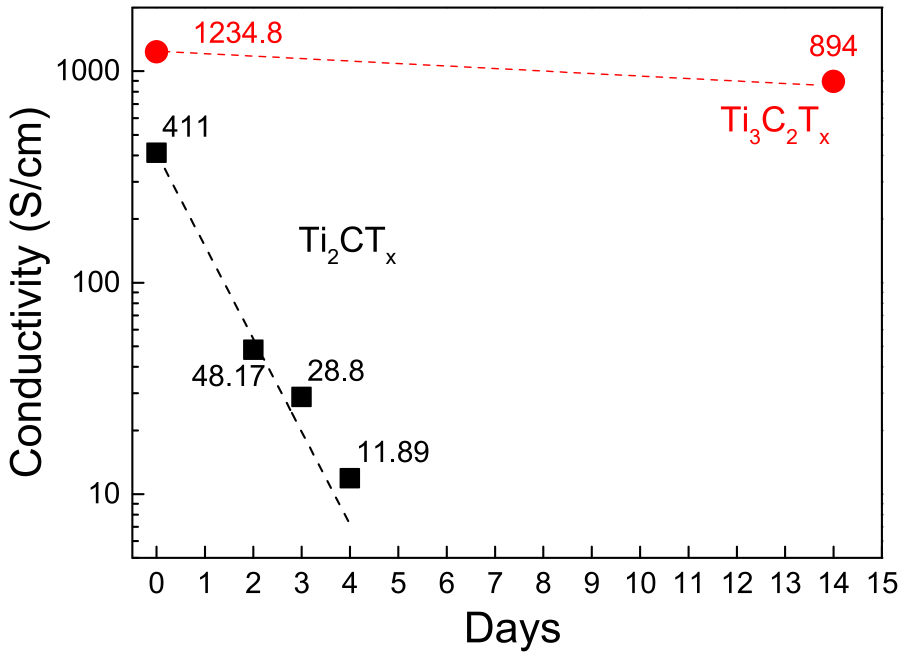

Fig. S6 Changes in the electrical conductivity of $Ti_3C_2T_X$ and Ti_2CT_X film over time when exposed to the atmosphere.

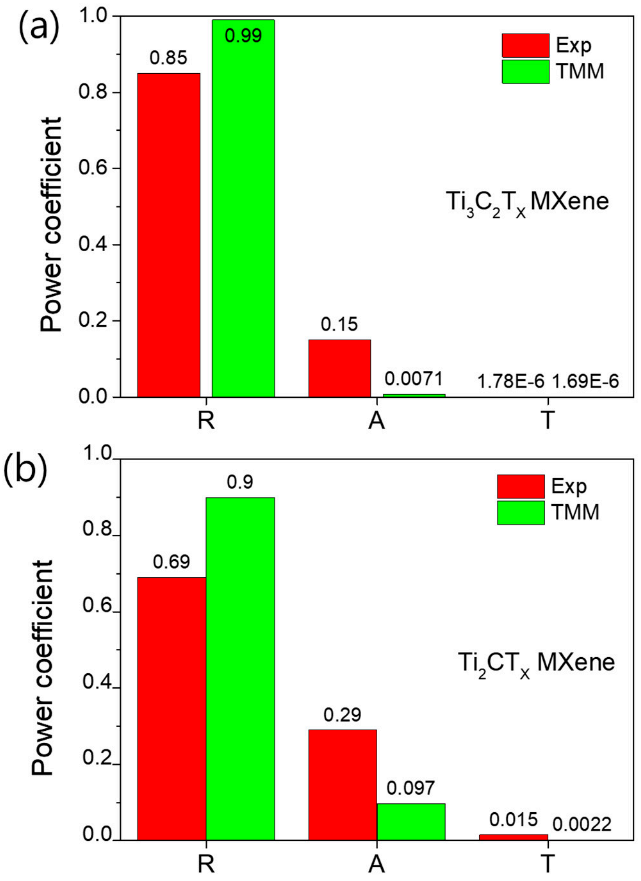

Fig. S7 (a), (b) Power coefficient, R, A and T, of $Ti_3C_2T_X$ and Ti_2CT_X film calculated with SE_R, SE_A and SE_T measured experimentally and calculated by transfer matrix method, respectively, using eqn. (1)-(3).
